# The lateral neocortex is critical for contextual fear memory reconsolidation

**DOI:** 10.1038/s41598-019-48340-9

**Published:** 2019-08-21

**Authors:** Verónica de la Fuente, Candela Medina, Germán Falasco, Leandro Urrutia, Alexxai V. Kravitz, Francisco J. Urbano, Silvia Vázquez, María Eugenia Pedreira, Arturo Romano

**Affiliations:** 1 Universidad de Buenos Aires, Facultad de Ciencias Exactas y Naturales, Departamento de Fisiología, Biología Molecular y Celular, Buenos Aires, Argentina; 2 CONICET-Universidad de Buenos Aires, Instituto de Fisiología, Biología Molecular y Neurociencias (IFIBYNE), Buenos Aires, Argentina; 3 Centro de Imágenes Moleculares, Fundación para la Lucha contra las Enfermedades Neurológicas de la Infancia (FLENI), Escobar, Buenos Aires, Argentina; 4 National Institute of Diabetes and Kidney and Digestive Diseases, Bethesda, MD 20814 USA

**Keywords:** Learning and memory, Neuroscience

## Abstract

Memories are a product of the concerted activity of many brain areas. Deregulation of consolidation and reprocessing of mnemonic traces that encode fearful experiences might result in fear-related psychopathologies. Here, we assessed how pre-established memories change with experience, particularly the labilization/reconsolidation of memory, using the whole-brain analysis technique of positron emission tomography in male mice. We found differences in glucose consumption in the lateral neocortex, hippocampus and amygdala in mice that underwent labilization/reconsolidation processes compared to animals that did not reactivate a fear memory. We used chemogenetics to obtain insight into the role of cortical areas in these phases of memory and found that the lateral neocortex is necessary for fear memory reconsolidation. Inhibition of lateral neocortex during reconsolidation altered glucose consumption levels in the amygdala. Using an optogenetic/neuronal recording-based strategy we observed that the lateral neocortex is functionally connected with the amygdala, which, along with retrograde labeling using fluorophore-conjugated cholera toxin subunit B, support a monosynaptic connection between these areas and poses this connection as a hot-spot in the circuits involved in reactivation of fear memories.

## Introduction

Consolidation is the process by which new information is encoded in neural circuits. However, most consolidated memories do not remain immutable indefinitely^1–3^; instead, they may change over time and with experience. Each time a reminder of the learning event is presented to an animal that has learned something new, the original memory can take distinct courses based on the specific characteristics of the reminder^4,5^. Specifically, a short re-exposure to the training context in rodents subjected to a contextual fear conditioning protocol triggers the expression of fear memory, and the trace may become labile and susceptible to disruption. The process of reconsolidation is needed for trace re-stabilization^6–8^, enabling changes in its strength^9–11^ and/or content^12,13^. In recent decades, labilization, reconsolidation and expression have been extensively studied using behavioral, cellular and molecular approaches in rodent models^14,15^. However, no *in vivo* whole-brain studies have been performed to elucidate the neural circuits and brain areas that subserve memory dynamics during these processes in small animals, despite its relevance in improving our understanding of fear-related dysfunctions in humans, such as phobias and post-traumatic stress disorders^16–18^.

Functional imaging techniques such as functional magnetic resonance imaging (fMRI) and positron emission tomography (PET) are very powerful tools to investigate brain areas involved in different tasks, primarily because of their minimal invasiveness^19^, and are widely applied in humans and non-human primates^20,21^. However, the use of these tools is uncommon in small animals. One previous study used small-animal PET to better understand fear memories^22^, but no distinctions were made regarding memory labilization, reconsolidation and expression.

In the present work, we studied the mouse brain from a functional perspective using small-animal PET and the radioactive tracer [18F]-FDG for the measurement of glucose uptake to identify brain areas involved in the labilization/reconsolidation of fear memory using a contextual fear-conditioning paradigm in mice. We found differences in glucose consumption in different regions including the temporal association cortex (TeA, also known as TEa), auditory areas (AUD), the perirhinal cortex (PER, also referred to as PRh or PERI), somatosensory cortex, hippocampus and amygdala in animals that underwent labilization/reconsolidation processes compared to animals that did not express the fear memory. The differences in glucose consumption revealed a marked temporal course of hyper- or hypo-consumption. Animals that only expressed but did not labilize/reconsolidate the memory trace exhibited significant differences from mice that both expressed the memory trace and underwent labilization and reconsolidation.

As many studies have addressed the hippocampal and amygdalar role in cognitive processes, we focused on the remaining areas TEa, AUD and PER. From now on, we use the all-encompassing term “lateral neocortex” to refer to these areas. We performed directed neuronal inhibition using *designer receptors exclusively activated by designer drugs* (DREADDs)^23,24^ and found that this brain region is necessary for reconsolidation. Using retrograde labeling and an optogenetic/electrode array-based strategy we demonstrate that the lateral neocortex projects to the amygdala, which is a key structure for the processing of emotional information^25^.

## Results

### Distinguishing between reminders that do or do not induce labilization/reconsolidation

**Figure 1:**
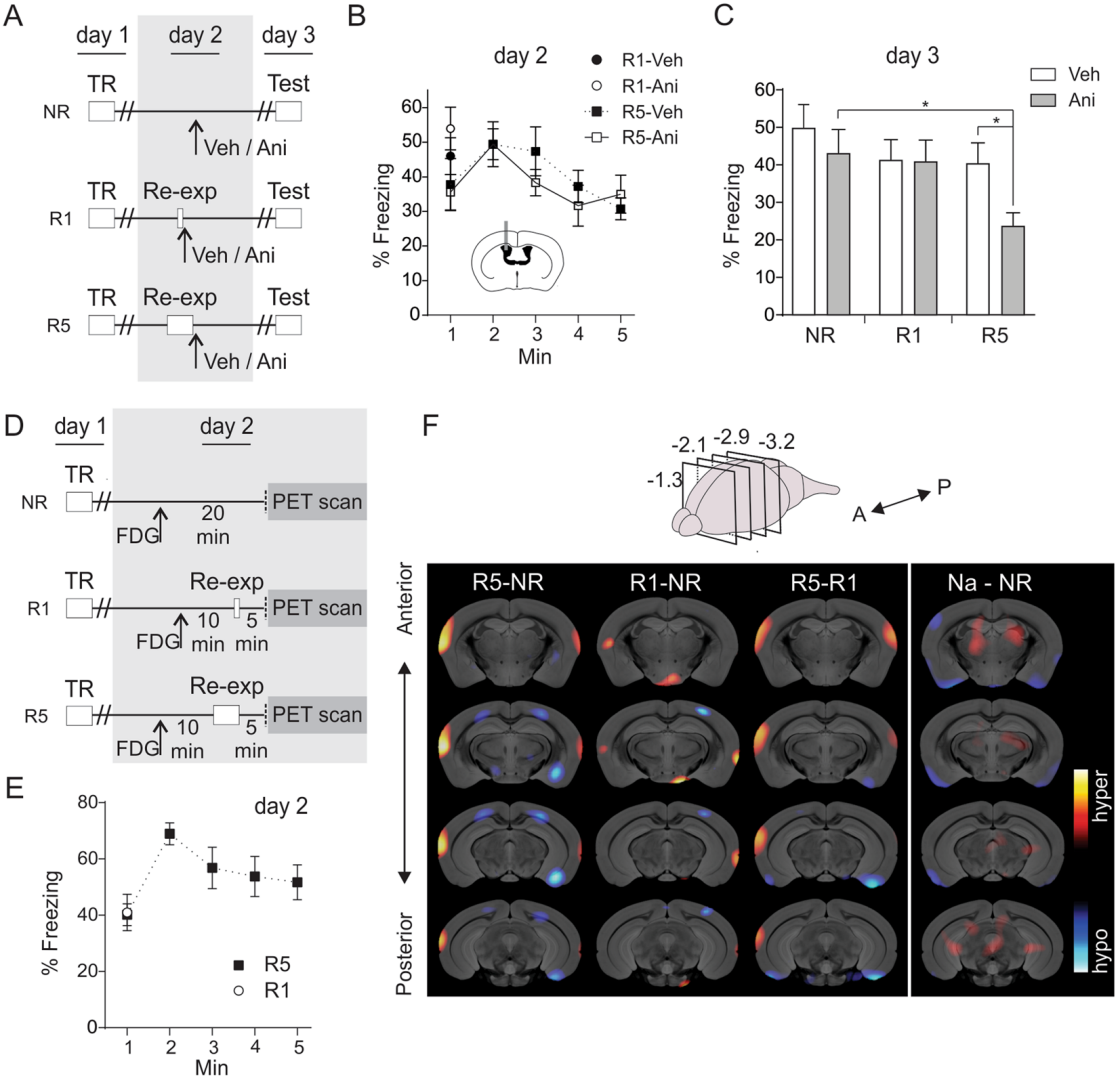
Differential glucose consumption in animals that express and labilizate/reconsolidate memory vs. animals that only express memory. (**A**) Contextual fear conditioning design. Trained mice were separated into three behavioral groups: non- re-exposed animals (NR), 1 min re-exposed animals (R1) and 5 min re-exposed animals (R5), and then further divided each behavioral group into two groups, according to the injection (Ani: Anisomycin or Veh: vehicle). We tested all mice for contextual memory on day 3. n = 13–15. TR: training; Re-exp: context re-exposure. Experimental procedures in day 2 are shown in grey. (**B**,**C**) Percentage of freezing on re-exposure and testing day, respectively. Data are shown as mean ± SEM. *P < 0.05. Inset shows ICV injection. (**D**) Experimental protocol, similar as in (**A**) but designed to measure glucose consumption. We injected mice [18F]-FDG IP 10 min pre re-exposure, and after re-exposure we anesthetized them with isoflurane and analyzed with PET (for simplicity both anaesthesia and acquisition of images with PET are altogether indicated as “PET scan”). Non-re-exposed mice were injected with [18F]-FDG 24 h post training. n = 11–12. (**E**) Percentage of freezing on re-exposure day. Data are shown as mean ± SEM. (**F**) Small-animal PET images corresponding to different coronal sections. The distance of each coronal section (in mm) relative to bregma is indicated in the upper scheme of the mouse brain (negative values for sections posterior to bregma). Data were analyzed using SPM ANOVA design and normalized through ANCOVA regressors. Comparisons yielding P values < 0.01 are shown using a t statistic color scale, which corresponds to the level of significance at the voxel level (detailed color scales in Supplementary Fig. 1). Images are displayed with the left side corresponding to the left hemisphere, according to neurological conventions.

Different reminders of a learning event may elicit different neural processes, and the behavioral output may take different courses depending on the characteristics of the reminder^4^. As the aim of this work was to study brain areas involved in memory labilization/reconsolidation using small-animal PET methodology and because when an animal expresses a memory, labilization/reconsolidation might also be elicited^26,27^, our first goal was to find protocols that would enable us to distinguish these latter memory processes from memory expression itself. Suzuki and co-workers used a fear-conditioning paradigm in rats and reported that a brief re-exposure (i.e., 3 min) to the training context one day after training elicited labilization/reconsolidation processes, whereas a shorter re-exposure of 1 min did not elicit these processes^5,28,29^. In line with these results, we trained ventricle-cannulated mice and separated the animals into 6 groups after 24 h. Two groups were re-exposed to the training context for 5 min, and half of these mice were injected with the protein synthesis inhibitor anisomycin (Ani; R5-Ani), which is a broadly used drug to induce memory impairment^30–33^. The other half of the mice received a vehicle solution (Veh; R5-Veh). Two other groups were re-exposed to the training context for only 1 min and subsequently injected with Ani or Veh (R1-Ani and R1-Veh, respectively). The last two groups were injected with Ani or Veh but not re-exposed (NR-Ani and NR-Veh, respectively). Memory was assessed 48 h after training (Fig. 1A). No differences in levels of freezing were observed in the first min of re-exposure in Ani vs. Veh injected mice (Fig. 1B; GLM: re-exposure effect: F_(1,54)_ = 4.92, P = 0.031; drug effect: F_(1,54)_ = 0.23, P = 0.63; interaction: F_(1,54)_ = 0.72, P = 0.40; contrasts (Sidak): R5-Ani vs. R5-Veh: F_(1,54)_ = 0.07, P = 0.96; R1-Ani vs. R1-Veh: F_(1,54)_ = 0.91, P = 0.57). However, only the R5-Ani group exhibited a long-term memory deficit on day 3, which demonstrated that a re-exposure of 5 min, but not 1 min, was sufficient to induce memory labilization and reconsolidation (Fig. 1C; GLM: re-exposure effect: F_(2,78)_ = 3.47, P = 0.036; drug effect: F_(1,78)_ = 2.94, P = 0.09; interaction: F_(2,78)_ = 1.25, P = 0.29; contrasts (Sidak): R5-Ani vs. R5-Veh: F_(1,78)_ = 5.79, P = 0.037; NR-Ani vs. R5-Ani: F_(1,78)_ = 7.32, P = 0.017).

### Memory reconsolidation and positron emission tomography

Having characterized the experimental conditions under which memory can be expressed or both expressed and labilized/reconsolidated, we performed an experiment similar to that illustrated in Fig. 1A but with the aim of analyzing local brain glucose consumption. We trained mice and the next day separated them into three groups (Fig. 1D; R5, R1 and NR). In the case of re-exposed animals, we administered [18F]-FDG intraperitoneally (IP) 10 min before the onset of a re-exposure session of either 5 or 1 min (R5 and R1, respectively). Five minutes after the mice were removed from the training context, we anesthetized them and PET images were acquired. We administered [18F]-FDG to non-re-exposed animals 24 h post-training and anesthetized them 20 min later. We chose the time of [18F]-FDG injection so that the behavioral moment of interest occurred in concordance with maximum radiotracer availability in brain plasma following IP injection^34^. Figure 1E shows that the freezing response of re-exposed groups was similar in the first min of re-exposure (GLM, F_(1,21)_ = 0.01, P = 0.91). Figure 1F shows the PET results (presence of color indicates significant differences with *P* values less than 0.01, analyzed using pair-wise comparisons; complete results in Supplementary Fig. 1). Among the various brain areas showing statistical differences, the lateral neocortex clearly showed higher glucose consumption in the R5 group than in the NR group, and this differential signal was stronger in the right side of the brain. Some hyper-consumption was observed in R1 compared to NR, but in the lateral neocortex, R5 consumed more glucose than R1. Strikingly, R5 exhibited a unilateral hypo-consumption in the amygdala compared to the NR group. Next, we evaluated whether the hyper-consumption observed in the lateral neocortex of the R5 group compared to NR group remained as hyper-consumption compared to naïve animals (Na). We performed another PET experiment to compare the glucose consumption of the Na and NR animals 24 h after TR. Pair-wise comparisons between these groups revealed significant differences in glucose consumption, but not in the lateral neocortex (Fig. 1F; Supplementary Fig. 1).

**Figure 2:**
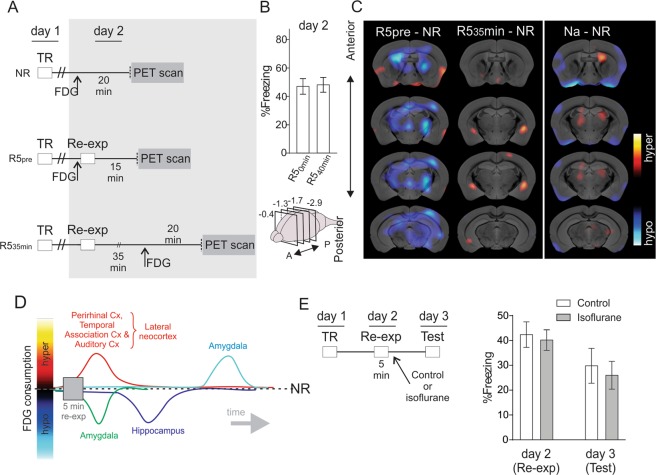
Dynamics of glucose consumption in animals that express and labilizate/reconsolidate memory. (**A**) Contextual fear conditioning design. We trained three groups of mice. The next day, we re-exposed two of them to the training context for 5 min and injected with [18F]-FDG, either pre re-exposure or 35 min after its offset (R5-pre and R5-35min, respectively). The third group was not re-exposed (NR) but received FDG injection 24 h after training and served for comparisons. Time interval between [18F]-FDG administration and anesthesia was maintained at 20 min (for simplicity, both anaesthesia and acquisition of images with PET are altogether indicated as “PET scan”). n = 12. TR: training; Re-exp: context re-exposure. Experimental procedures in day 2 are shown in grey. (**B**) Percentage of freezing on re-exposure day. Data are shown as mean ± SEM. (**C**) Small-animal PET images corresponding to different coronal sections. The distance of each coronal section (in mm) relative to bregma is indicated in the upper scheme of the mouse brain (negative values for sections posterior to bregma). Data were analyzed using SPM ANOVA design and normalized through ANCOVA regressors. Comparisons yielding P values < 0.01 are shown using a t statistic color scale, which corresponds to the level of significance at the voxel level (detailed color scales in Supplementary Fig. 2). Images are displayed with the left side corresponding to the left hemisphere, according to neurological conventions. (**D**) Schematic temporal course of glucose consumption after 5 min re-exposure to the training context, summarizing results shown in Figs. 1 and 2 (Cx: cortex). (**E**) Anaesthesia (isoflurane) after retrieval does not affect long-term fear memory. Left panel: Contextual fear conditioning design. We trained two groups of mice. The next day, we re-exposed mice to the training context for 5 min and exposed one group to isoflurane as previously done in experiments in Figs. 1A and 2A, while the other group remained as control. Both groups were tested for long-term memory 24 h after re-exposure to the training context. Right panel: Percentage of freezing on re-exposure and testing days. Data are shown as mean ± SEM. n = 9–10. TR: training; Re-exp: context re-exposure.

We administered [18F]-FDG at different time-points, taking the offset of re-exposure session as time zero, to identify the temporal course of local brain glucose consumption after exposure to specific contextual stimuli that elicit labilization/reconsolidation (Fig. 2A). Animals were re-exposed to the training context for 5 min 24 h after the training session. We administered [18F]-FDG immediately prior to the re-exposure session to one group of animals (R5-pre) and 35 min after removal from the conditioning chamber to another group of animals (R5-35 min). A third group of animals was non-re-exposed (NR), injected with [18F]-FDG 24 h after the training and used for comparisons. Both re-exposed groups froze at similar levels during the re-exposure session (Fig. 2B; GLM, F_(1,22)_ = 0.02, P = 0.89). Pair-wise comparisons against the NR group indicated that the R5-pre mice exhibited a widely dispersed hypo-consumption in the brain, including the hippocampal areas (Fig. 2C, left; Supplementary Fig. 2). The R5-35 min mice exhibited a bilateral hyper-consumption in the amygdala (Fig. 2C, middle; Supplementary Fig. 2). Naïve mice did not exhibit differences in the aforementioned brain areas compared to the NR mice (Fig. 2C, right; Supplementary Fig. 1).

We also investigated glucose consumption in 1-min re-exposed mice injected with [18F]-FDG 35 min after removal from the conditioning chamber (R1-35 min) to assess whether amygdalar hyper-consumption was related to the expression of memory itself or the labilization/reconsolidation processes. Pair-wise comparisons against NR animals revealed significant differences but not in the brain areas noted above (Supplementary Fig. 3).

One caveat of studying memory in animals that are treated with anesthesia is that, depending on the behavioral experiment, it might affect the memory trace differently. In this sense, anesthesia might have affected the memory trace in animals that were re-exposed to the training context and not to non-re-exposed animals (NR) or non-labilized animals (R1), because the former group is the one in which the memory trace gets labile. To assess whether anesthesia after memory reactivation affects the behavioral output, we performed an experiment comparing freezing behavior in two groups of trained mice. One group was anaesthetised with isoflurane after a 5 min re-exposure to the training context (R5-isoflurane, n = 10) and the other group was not anaesthetised after re-exposure (R5-control, n = 9; Fig. 2E left). Freezing was calculated both in re-exposure (24 h after training) and testing day (24 h after re-exposure). No differences were observed neither in the re-exposure day nor in the testing day (Fig. 2E right; repeated-measures GLM: day effect: F_(1,17)_ = 18.01, P = 0.0005; drug effect: F_(1,17)_ = 0.18, P = 0.684; interaction: F_(1,17)_ = 0.07, P = 0.80; contrasts (Sidak): day 2 R5-control vs. R5-isoflurane: F_(1,17)_ = 0.11, P > 0.99; day 3 R5-control vs. R5-isoflurane: F_(1,17)_ = 0.18, P > 0,99).

Altogether, 5-min re-exposed mice exhibited differences in local brain glucose consumption compared to NR mice at various time-points, thus tracing a temporal course of the effects elicited by the stimuli. Within this study, the lateral neocortex, amygdala and hippocampus each exhibited differential glucose consumption at one or more of the time-points measured (Fig. 2D).

### The lateral neocortex is necessary for fear memory reconsolidation

Our PET studies identified brain areas that were not previously implicated in contextual fear labilization/reconsolidation processes (encompassed here as the lateral neocortex). Therefore, we further examined the role of these areas using a functional study. We used a DREADD-based strategy^24^ to control neurons in the lateral neocortex *in vivo*, given that the designer drug clozapine N-Oxide (CNO) can be administered IP^35^ and not in a specific brain area, which would obscure PET results due to possible glucose consumption related to cannula implantation. This technique would allow the measurement of both behavioral output and glucose consumption using the same methodologies, with the advantage of neuron manipulation in specific brain areas and time windows. The chosen viral vector expresses both hM4D, which is an engineered version of the M4 inhibitory muscarinic acetylcholine receptor that lacks an endogenous ligand but is sensitive to the drug CNO^36^, and the fluorescent protein mCherry under the control of the CamkIIa promoter in the intended injection sites. We hypothesized that CNO administration near the reconsolidation time window would impair long-term memory if the lateral neocortex was important for the occurrence of that memory process.

#### Electrophysiological validation of DREADDs

**Figure 3:**
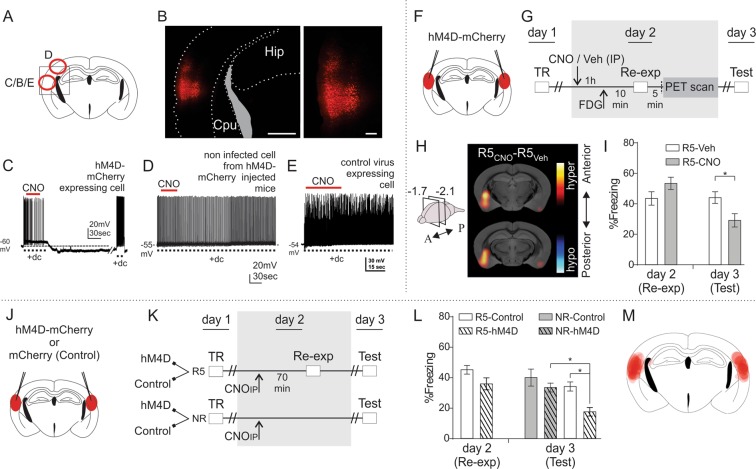
The lateral neocortex is necessary for contextual fear memory reconsolidation. (**A**) Schematic coronal view of a mouse brain at −2.1 mm posterior to bregma, indicating with circles the sites for patch clamp of cells shown in (**C**–**E**). The square indicate site for the image shown in B. (**B**) Morphologic validation of construct expression. Left: fluorescence microscopy image of CamkIIa-hM4D-mCherry expressing mouse showing extension of viral infection. Scale bar: 1 mm. Hip: hippocampus; CPu: caudate-putamen; In grey: lateral ventricle. Right: scale bar: 0.2 mm. (**C**) Representative Whole Cell Current Clamp recording of a fluorescent cell from a mouse injected in the lateral neocortex with the viral vector expressing CamkIIa-hM4D-mCherry. (**D**) Idem (**C**) but for a non-fluorescent cell of the primary somatosensory cortex. (**E**) Idem C but from a mouse injected with control virus CamkIIa-mCherry. CNO local puff (100 µM). +dc: depolarizing current. (**F**) Schematic coronal view of a mouse brain at −2.1 mm posterior to bregma, indicating sites of bilateral injection of the viral vector expressing CamkIIa-hm4D-mCherry in the lateral neocortex. (**G**) Contextual fear conditioning design. Mice expressing h m4D-mCherry in the lateral neocortex were trained and the next day were re-exposed for 5 min to the training context. Previous to re-exposure, some mice were injected with CNO IP while others were injected with vehicle solution (Veh). All mice received [18F]-FDG IP 10 min pre re-exposure (time point as in Fig. 1D). After re-exposure mice were anesthetized and PET images were acquired (for simplicity, both anaesthesia and acquisition of images with PET are altogether indicated as “PET scan”). Contextual fear memory was tested on day 3. n = 12–13. (**H**) Small-animal PET images corresponding to coronal sections where glucose consumption statistical differences were found. The distance of each coronal section (in mm) relative to bregma is indicated in the left scheme of the mouse brain (negative values for sections posterior to bregma). Data were analyzed using SPM ANOVA design and normalized through ANCOVA regressors. Comparisons yielding P values < 0.01 are shown using a t statistic color scale, which corresponds to the level of significance at the voxel level. Images are displayed with the left side corresponding to the left hemisphere, according to neurological conventions. (**I**) Percentage of freezing on re-exposure day (day 2) and testing day (day 3). Data are shown as mean ± SEM. *P < 0.05. (**J**) Similar as in (**F**), except that mice were either injected with viral vectors expressing hM4D-mCherry or mCherry alone (control). (**K**) Contextual fear conditioning design. All mice were trained, and half of the mice expressing hM4D-mCherry were re-exposed for 5 min to the training context (R5-hM4D) while the other half was not (NR-hM4D). The same was done for Control mice (R5-Control and NR-Control groups). Contextual fear memory was tested on day 3. n = 10–12. Percentage of freezing on re-exposure day (day 2) and testing day (day 3). Data are shown as mean ± SEM. *P < 0.05. (**M**) Schematic representation illustrating viral injection sites; opacity correlates with number of mice expressing CamkIIa-hM4D-mCherry or CamkIIa-mCherry in a given location.

We first injected mice in the lateral neocortex with viral vectors carrying hM4D (CamkIIa-hM4D-mCherry) or the control construction (CamkIIa-mCherry). Fluorescence indicative of mCherry expression was examined 2 weeks later to confirm viral expression (Figs. 3A,B). We also performed electrophysiological recordings in acute slices to examine the functionality of the expressed receptor hM4D. Patch clamp recordings on fluorescent cells from CamkIIa-hM4D-mCherry injected mice indicated that these cells elicited a train of action potentials upon stimulation and that puff-applied CNO silenced these neurons and hyperpolarized the cells afterwards (Fig. 3C). In contrast, CNO did not affect the firing frequency of a non-fluorescent cell from the somatosensory cortex (Fig. 3D) or a fluorescent cell from control viral vector-injected animals (Fig. 3E).

#### PET, DREADDs and behavior

We then assessed the effect of lateral neocortex inhibition on memory reconsolidation based on the results shown in Fig. 1. This experiment would also enable us to confirm the proper functional inhibition, which would be indicated by differential glucose consumption in target areas. Two weeks after bilateral injection of the CamkIIa-hM4D-mCherry viral vector, mice were trained, and CNO or vehicle solution (Veh) was injected IP one day after training. One hour later, mice were injected with [18F]-FDG and placed in the training context 10 min later for 5 min (R5-CNO and R5-Veh groups). Mice were anesthetized 5 min after removal from the conditioning chamber, and PET images were acquired. We assessed memory 24 h after re-exposure (i.e., 48 h after training; Figs. 3F,G). The R5-CNO and R5-Veh groups did not differ in glucose consumption in the targeted brain area (Fig. 3H; presence of color indicates significant differences with *P* values less than 0.01, analyzed using pair-wise comparisons). Two hypotheses may explain these results: no inhibition following CNO administration or inhibition of viral-infected cells, which was not detectable using this imaging technique. Notably, the amygdala was the only brain area that exhibited differences in glucose consumption between groups. The difference was bilateral, albeit greater on one side. In other words, inhibition of the lateral neocortex within a temporal window in which mice were re-exposed to the training context revealed that the amygdala consumed more glucose in the experimental group than in control mice. Regarding the effects on behavior, on day 2, the R5-CNO group showed a froze at levels similar to those of the R5-Veh group, while on day 3 the former group exhibited memory deficits (Fig. 3I; repeated-measures GLM: day effect: F_(1,23)_ = 19.71, P = 0.002; drug effect: F_(1,23)_ = 0.11, P = 0.74; interaction: F_(1,23)_ = 21.32, P = 0.001; contrasts (Sidak): day 2 R5-CNO vs. R5-Veh: F_(1,23)_ = 4.89, P = 0.073; day 3 R5-CNO vs. R5-Veh: F_(1,23)_ = 7.68, P = 0.022). The substantial rise in amygdalar glucose consumption and the CNO-elicited long-term memory deficit support the second hypothesis. This experiment also demonstrated that hM4D expression itself did not account for the behavioral output observed.

We performed a DREADD-based experiment including non-re-exposed (NR) animals as a control group to confirm whether the inhibition of the cortex of interest specifically affected the memory reconsolidation process. The CamKIIa-hM4D-mCherry viral vector or the control vector, CamKIIa-mCherry, was injected to examine the effect of the type of virus injected instead of the drug administered (Figs. 3J,K,M). Behavioral conditioning was performed 2 weeks after surgery. We trained all mice in the fear-conditioning paradigm, and mice injected with the control vector were separated into two groups the next day. One group was re-exposed to the training context for 5 min (R5-Control), and the other group was non-re-exposed (NR-Control). Mice injected with the hM4D vector were divided in a similar manner (R5-hM4D and NR-hM4D). All re-exposed mice received CNO IP 70 min prior to re-exposure to the training context (the same moment as in the experiment in Fig. 3G). Non-re-exposed animals were injected with CNO 24 h after training. Memory was assessed 24 h after re-exposure (for NR animals, 48 h after training). Both re-exposed groups froze at similar levels on day 2 (Fig. 3L; GLM, F_(1,21)_ = 3.81, P = 0.064). However, re-exposed mice expressing hM4D in the cortex of interest exhibited lower levels of freezing than re-exposed mice expressing the control construct on day 3. These mice also exhibited lower levels of freezing than non-re-exposed mice expressing hM4D in the lateral neocortex (GLM: re-exposure effect: F_(1,41)_ = 9.86, P = 0.003; type of virus effect: F_(1,41)_ = 11.54, P = 0.001; interaction: F_(1,41)_ = 14.59, P = 0.0004; contrasts (Sidak): R5-Control vs. R5-hM4D: F_(1,41)_ = 26.72, P < 0.0001; R5-hM4D vs. NR-hM4D: F_(1,41)_ = 24.85, P < 0.0001; NR-hM4D vs. NR-Control: F_(1,41)_ = 0.09, P = 0.99). These latter experiment demonstrates that CNO itself is not an amnesic agent, which is a plausible hypothesis based on the results in Fig. 3I. Therefore, the injection of CNO *together with* expression of the hM4D receptor sufficiently explain the results.

### Amygdala receives monosynaptic connections from the lateral neocortex

**Figure 4:**
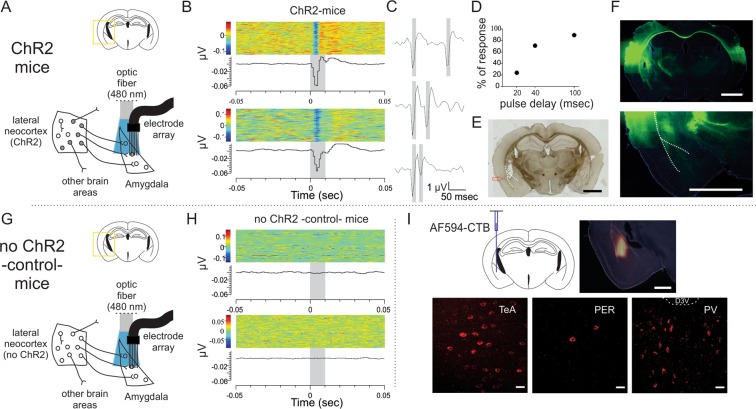
Monosynaptic connection between the lateral neocortex and the amygdala. (**A**) Experimental design for items B-F. We injected ChR2 expressing viral vector in the lateral neocortex and implanted optic fiber plus movable-15 electrode array in amygdala. (**B**) Local field potential responses to 10 msec light pulses (480 nm) in ChR2 expressing mice (n = 4). Two electrode with responses are shown. Each of them shows a raster plot (above) and a histogram (below) (1 msec bins). (**C**,**D**) Paired pulse depression is observed when decreasing pre pulse interval, supporting a pre-synaptic effect. (**E**) Placement of the tip of the cannulae containing the movable electrodes (Scale bar: 2 mm). (**F**) Fluorescence microscopy images showing ChR2-YFP expression. Scale bars: 2 mm. Note that no somatic expression is seen in amygdala. (**G**) Same as in (**A**) except that mice did not expressed ChR2 -control- (n = 2). (**H**) Same as in (**B**) but using control mice. (**I**) Alexa Fluor 594 CTB (AF594-CTB) injection in amygdala (upper; Scale bar 1 mm) rendered red-somas in the lateral neocortex (lower; scale bar 30 µm), particularly in the TeA, PER, and paraventricular thalamic nucleus (PV).

Our results provided evidence of a functional connection between the lateral neocortex and the amygdala in PET analysis, which revealed that inhibition of the lateral neocortex altered glucose consumption levels in the amygdala (Fig. 3H). Therefore, we investigated the synaptic connections supporting these results. We used an optogenetic-based strategy in which we expressed the light-activated cation channel rhodopsin (ChR2)^37,38^ in the lateral neocortex on the left side of the brain and implanted an electrode array plus optic fiber in the amygdala in this hemisphere. If these regions were connected, then light stimulation into the amygdala would excite ChR2-expressing axons reaching from the lateral neocortex, and a significant change in amygdalar local field potential (LFP) responses would be measured (Figs. 4A–F). Light-evoked responses were observed on 54% of the electrodes in the 4 mice tested, with a maximum peak mean latency of 6.3 msec (peri-event raster plots of two electrodes with light-evoked responses are shown in Fig. 4B). We further performed paired-pulse stimulation to vary the interval between light pulses and observed a deflection of the second pulse amplitude when the pulse delay was diminished (Figs. 4C,D), indicating monosynaptic connectivity between the lateral neocortex and amygdala. No light-evoked responses were observed in control mice that did not express ChR2 (Figs. 4G,H), which excludes the possibility of a light artifact. The monosynaptic connection was also assessed morphologically. The retrograde tracer cholera toxin subunit B (CTB) conjugated to the fluorophore Alexa Fluor 594 (AF594-CTB) was injected into the amygdala, and fluorescent cells were observed in the TeA, PER and other areas known to project to the amygdala such as the paraventricular thalamic nucleus^39–41^ (PV; Fig. 4I).

## Discussion

In the present study we used a new approach to investigate whole-brain memory dynamics using small-animal PET scanning to examine the labilization and reconsolidation phases of memory. This approach revealed that some brain areas consumed glucose differently in animals that underwent labilization and reconsolidation. A broad brain zone comprising TeA, AUD and PER, termed here *lateral neocortex*, were part of those areas and its in-deep study became relevant as the knowledge of their roles in the reactivation of fear memory processes is scarce. A DREADD-based strategy revealed the lateral neocortex is necessary for fear memory reconsolidation. Morphological and functional evidence of projections from the lateral neocortex to the amygdala was also demonstrated, supporting previous results obtained in rats^42–44^ and posing these circuits as a hot-spot involved in reactivation of fear memories.

It is generally believed that labilization and reconsolidation occur when memory is expressed. However, pharmacological studies support the independence of these processes. For instance, impairment of expression does not prevent labilization and reconsolidation in conditioned taste aversion^45,46^ or novel object recognition in rodents^47,48^, and in the context-signal memory model in the crab *Neohelice granulata*^49^. Similar results were observed for memory extinction^50^. In our experiments we included a group that only expressed the memory (R1) to distinguish labilization/reconsolidation from memory expression and observed differences in local brain glucose consumption between the R1 group and the group that also underwent labilization and reconsolidation (R5), which supports the hypothesis that the areas that exhibited significant differences were differentially involved in labilization/reconsolidation processes. However, the R5 group also expressed the memory for longer time periods, which makes it impossible to completely exclude an effect of a longer expression. The DREADD-based experiments allowed us to assess this potential effect: levels of freezing during re-exposure were similar in control and experimental groups despite being under the expected effect of CNO^35^, which strongly suggest that the effects observed in the present work were due to labilization/reconsolidation processes.

### Brain sites involved in contextual fear memory reconsolidation

Different parts of the brain are relevant for the reconsolidation of particular types of memory depending on the learning paradigm. The hippocampus and the amygdala are the most-studied brain areas for contextual fear memories because of their importance in encoding contextual information and emotions, respectively^51^. Other cortical brain areas are also necessary for contextual memory reconsolidation, primarily the pre-limbic subregion of the medial prefrontal cortex (PL-mPFC)^52–54^, the anterior cingulate cortex (ACC)^55,56^, and the entorhinal cortex^51,57^. In our experiments, we affected reconsolidation of a contextual fear memory through chemogenetic inhibition of a brain area comprising mainly the TeA and AUD, with the PER in a lesser extent (Fig. 3). Information about the role of these three areas in fear reconsolidation, especially in contextual-based conditionings, is scarce. In regard to the TeA, it has been shown that it (together with AUD according to histology) is necessary for remote cued memory recall, and that different genes are expressed after cued fear conditioning^58^. Concerning the PER, previous studies assessed its role in reconsolidation of both cued and contextual fear memories. Regarding contextual fear memories, lesions in PER at different time-points after training affects memory expression^59^; CA1 engram cells inhibition affects expression and diminishes PER activation^60^. Another study performed in cued fear conditioning found that TTX inactivation of PER after recall did not affect reconsolidation^61^. These latter results, albeit performed using cued- shock associations, contradict our findings. However, the differences might be attributed to the timing of drug administration. With respect to AUD role in reconsolidation of fear memory, using a cued based paradigm it has been shown that AUD is activated after retrieval^62,63^ and AUD lesion affects memory expression^64^. Considering our results showing AUD relevance in contextual fear reconsolidation, further investigations are needed to fully understand its possible integrative role in this type of memory trace. It is worth to note that AUD refers to several areas, such as the primary auditory cortex, the ventral and dorsal secondary auditory cortex, which differ in afferents and efferents and have different roles in processing and integration of information^42^. Our PET results and DREADDs expression cannot distinguish between them but for sure includes secondary ventral auditory cortex.

In fact, a limitation of our study is the resolution of the PET methodology. The areas PER, TeA and AUD are physically very close to each other, so it is still not known whether the three of these areas, some of them or even smaller regions of each are necessary for contextual fear reconsolidation. Therefore, this technique could be well complemented with others with cellular resolution, such as immediate-early genes (IEGs) activation mapping. Combining both approaches would enable a deeper understanding of the neuronal processes, as PET provides the advantage of studying activation patterns in an intact, living brain and IEGs mapping allows to improve their resolution in fixed tissue. It is worth to note that the PET experiments were performed to evaluate cumulative glucose consumption within a brief period of time (from [18F]-FDG administration to image acquisition) at different times after the beginning of the re-exposure session (see Results section). Therefore, other brain areas with known involvement in the process (see above) may have been overlooked in this study.

Previous work used small-animal [18F]-FDG-PET scanning to examine areas that are differentially involved in expression of contextual and cued conditioning^22^. The authors analyzed glucose consumption during fear memory testing on three groups of rats: One that had been trained using a pairing of tones and shocks (“FEAR” group), other which had been trained using tone and shocks in an unpaired scheme (“ANX” group), and a third control group (“CTRL”) which had only been exposed to the tones in the training context. The authors compared the ANX and CTRL groups and the ANX and FEAR groups respectively and observed a hyper-metabolism in brain areas that comprised the bed nucleus of the stria terminalis (BST) in the ANX group in both comparisons, which provided new evidence for a role of the BST in the expression of contextual memory. The ANX group is similar to our R5 group in the elicitation of contextual fear memory expression. However, it is worth noting that their testing parameters may also have elicited labilization/reconsolidation processes. The use of un-shocked (CTRL) animals as a control for comparisons makes it difficult to determine whether ANX-induced differential consumption after testing was due to consolidation-related processes, expression, or labilization/reconsolidation. The inclusion of a non-re-exposed trained group (termed NR) in our experiments excludes the possibility of consolidation-related mechanisms as an explanation of our results.

### Glucose consumption, PET and memory processes

Regarding glucose consumption, it is interesting to analyze its underlying biological meaning as PET provides a 3D map of [18F]-FDG local consumption but does not identify the cellular components of the local areas that are metabolically active. Despite the growing knowledge about glia importance in brain metabolic processes^65^ and cognition^66,67^, together with the increasing use of [18F]-FDG in PET for both clinical studies and basic research, it still remains controversial which cell types contribute to the [18F]-FDG PET signal^68,69^. Zimmer *et al*. recently reported direct evidence that activation of astrocytic glutamate transport via the excitatory amino acid transporter GLT-1 triggered a widespread but graded glucose uptake in the rodent brain using PET and claimed that astrocytes should be recognized as contributing to the [18F]-FDG signal^70^.

New learning, along with the further processing of information, initiates a cascade of cellular events that require high metabolic demands (e.g., gene expression, protein synthesis, and neuronal firing). Therefore, our initial hypothesis was that mice that underwent memory reprocessing after retrieval (i.e., reconsolidation) would exhibit greater glucose consumption than mice that did not. However, PET results revealed not only hyper-consumption but also hypo-consumption of glucose. Notably, the hypo-consumption appeared in brain regions known to be involved in the reconsolidation phase of memory, such as the hippocampus and amygdala, according to different studies that investigated the need of energy-demanding processes, such as gene transcription and protein synthesis^71–73^. However, hypo-signals are not unusual in healthy brain studies. Luyten *et al*. performed small-animal [18F]-FDG-PET studies and found hypo-consumption in areas critical for emotional behavior, including the amygdala^22^. The simultaneous consideration of all of the different players in the brain (excitatory/inhibitory neurons and glia) acting in concert during animal behavior is important to further elucidate hyper- and hypo-consumption. The PET final glucose consumption signal is related to the balance of these metabolically active cells.

### Looking forward

Cognitive function is conceived as a product of the concerted and synchronized activity of many brain areas in a hierarchical relation, with a low number of highly connected brain regions or nodes (hubs) and a high number of lower connected nodes^74,75^. Despite this network comprehensive concept of the brain, few studies have used brain-wide techniques to understand brain function in small animals and most of them have relied on fixed tissue to do so^76–80^ but see^81^. Here, we provide new insights in the study of the dynamics of the activation of brain areas during the reprocessing of the mnemonic trace due to behavioral experience using a novel technique for the field, small-animal PET. Understanding the processes elicited by memory retrieval is important for its theoretical value and provides insight into pathologies related to memory dysfunctions to aid in the development of therapeutic strategies^14,16,17^. Brain wide-techniques provide new information on areas involved in cognitive processes in a dynamic manner and help tackle the challenge of investigating cognitive functions by simultaneously considering the coordinated activity of multiple brain regions.

## Materials and Methods

### Animals

Male C57BL/6 mice 8–12 weeks old, weighting 25–30 g (La Plata University animal facilities, La Plata, Argentina; NIH, USA) were housed in groups of 4–5 except for cannulated/implanted mice, which were individually housed. In all cases, they were provided with water and food *ad libitum* under a 12 h light/dark cycle (lights on at 8:00 A.M.) at a temperature of 21–23 °C. Experiments were performed during the light cycle (between 9 A.M. and 4 P.M.) and were designed and performed with the approval of the University of Buenos Aires Institutional Animal Care and Use Committee (CICUAL N°29/2014) and in accordance with regulations of the National Institutes of Health (NIH) *Guide for the Care and Use of Laboratory Animals* (NIH publication 80–23/96), USA. We made all efforts to minimize animal suffering and to reduce the number of animals used.

### Behavioral procedures

Before training, the animals were handled once a day for two days. The training session consisted of placing each mouse in the conditioning chamber and allowing two min adaptation period after which it received three foot-shocks (0.6 mA, 1 s) separated by an interval of 1 min. After the final foot-shock, the mouse remained in the chamber for an additional min and returned to its home cage. One day after training the mice were re-exposed to the training context without presenting foot-shocks for either 5 or 1 min, or were no re-exposed at all. Further contextual test was performed 48 h after training by placing the mice in the training environment for 5 min in the absence of the foot-shock. Each session was videotaped to calculate freezing, defined as the absence of all movements except those related to breathing, and scored blindly according to an instantaneous time-sampling procedure in which each animal was observed every 5 s in the whole length of each session. Memory was assessed and expressed as the percentage of time that mice spent freezing, which is commonly used as an index of fear in rodents^82^. In specified experiments, PET analysis was performed in the re-exposure day. For details on the conditioning chamber see^83^.

We consider memory expression when the experimenters detect what has been specified as the operative measure of memory (in this case, freezing), that is, memory expression refers to behaviour. We consider memory labilization as the circuital changes that makes the trace labile, and is elicited under the presentation of certain types of reminders. Reconsolidation, in turn, is the process that re-stabilizes that trace. In PET studies we will not be able to differentiate between both processes, and thus the changes seen will be attributable to both. However, in pharmacological experiments in which the operative measure of memory is lower than in controls, we will assign the effect to a reconsolidation disruption (e.g. with anisomycin or with chemogenetic-mediated inhibition of neurons). When labilization is disrupted, on the contrary, the effect would be to see no changes in freezing (memory does not became labile, and thus cannot be disrupted). The term reactivation will be used to refer to the circuital process that allow labilization/reconsolidation, and not to the circuit process subserving memory expression.

### Cannulae implantations

For ICV injections, mice were deeply anesthetized (100 mg ketamine and 10 mg xylazine per kg. body weight co-injected IP) and placed in a stereotaxic frame. After exposing the skull, one 23 gauge guide cannula was implanted dorsally to either the left or right ventricle at coordinates: −0.2 mm anteroposterior from bregma; 1 mm lateral from midline; −1.2 mm ventral from skull surface; in accordance with the atlas of Franklin and Paxinos^84^ and personal adjustments. Guide cannulae were fixed to the skull with dental acrylic containing calcium hydroxide. While anesthetized, mice received one dose of antibiotic (enrofloxacin, 85 mg/kg) co-injected with analgesic SC (meloxicam, 5 mg/kg), and after surgeries were administered PO with analgesic (tramadol, 20 mg/ml, in the water bottle). Experiments were performed following animal recovery and drug injections were administered without anaesthesia. The injection device consisted of a 30 gauge cannula connected to a 5 µl Hamilton syringe by tubing. Initially, the injection device was filled with distilled water and a small air bubble was sucked into the injection cannula, followed by the injection solution. The air bubble allowed for visual inspection of the injection progress. The injection cannula was inserted into the guide cannula with its tip extending beyond the guide by 1 mm in order to reach the aimed zone. The injections were administered during 30 s and operated by hand. The injection cannula was removed after 60 s in order to avoid reflux and to allow the diffusion of drugs. The volume of each injection was 1 µl. Different injection devices were used for drug and vehicle. To verify cannulae placement, after behavioral procedures the animals were killed and their brains were placed in 4% paraformaldehyde (PFA) for one day followed by 30% sucrose in PBS for an additional 24 h. Brains were sliced using a vibratome and analyzed with a magnifying glass. Only data from animals with cannulae located in the intended sites were included in the analysis.

### Chemogenetics

For viral injections, we anesthetized the mice as mentioned before and bilaterally injected an anterograde adeno-associated viral vector (AAV5) carrying a cre-recombinase independent hM4D fused to mCherry, under the control of the excitatory neuronal promotor CamkII or control vector (AAV5-CamkIIa-hM4D-mCherry and AAV5-CamkIIa-mCherry, respectively; ~0.25 µl/side; University of North Carolina Vector Core) into the lateral neocortex, using microcapillary calibrated pipettes and a Picospritzer II (Parker Hannifin Corp). Coordinates were: 2.1 mm anteroposterior from bregma; ±4.5 mm lateral from midline; 1.3 mm ventral from brain surface (2° angled towards the left, in the left injection and 2° towards the right, in the right injection) in accordance with the atlas of Franklin and Paxinos^84^ and personal adjustments. Behavioral and PET acquisition were performed two weeks after viral injections. Mice received a single IP CNO injection (see Results). After experiments finished, mice were terminally anesthetized with ketamine/xylazine and transcardially perfused first with saline and then with 4% PFA. Brains were post-fixed in PFA ON and remained in PBS until used. Coronal sections were prepared for fluorescence analysis to check viral expression in the intended sites.

### Drugs

Anisomycin (Sigma, A9789) was first dissolved in acidic saline, then taken to pH ~7.5; and was injected ICV at 0.1 mg/µl. CNO (NIH) was administered IP at 5 µg/g^35^. It was first dissolved in DMSO, then in saline (final DMSO 2.5%). Timing of CNO administration was based on previous literature in order to achieve the greatest neuronal inhibition^35^ during the expression of memory.

### Whole-cell patch-clamp recordings *in vitro* using brain coronal slices

Mice were deeply anesthetized with tribromoethanol (250 mg/kg; IP) followed by transcardial perfusion with ice-cold N-methyl-D-glucamine based artificial cerebrospinal fluid (NMDG-aCSF) (composition in mM: 92 NMDG, 2.5 KCl, 1.25 NaH_2_PO_4_, 30 NaHCO_3_, 20 HEPES, 25 glucose, 2 thiourea, 5 Na-ascorbate, 3 Na-pyruvate, 0.5 CaCl_2_· and 10 MgCl_2_. pH was adjusted to 7.3–7.4 with concentrated hydrochloric acid, and aerated with 95% O_2_/5% CO_2_), and then decapitated. Coronal brain slices (300 μm) were obtained gluing both hemispheres onto a vibratome aluminum stage (Integraslicer 7550 PSDS, Campden Instruments, UK), submerged in a chamber containing NMDG-aCSF. Slices were sequentially cut and transferred to an incubation chamber containing NMDG-aCSF at 35 °C for 30 min and then transferred to a second chamber containing low Ca^2+^/high Mg^2+^ normal aCSF (composition in mM: 125 NaCl, 2.5 KCl, 3 MgSO_4_, 0.1 CaCl_2_, 1.25 NaH_2_PO_4_, 0.4 ascorbic acid, 3 myo-inositol, 2 pyruvic acid, 25 d-glucose, and 25 NaHCO_3_ and aerated with 95% O_2_/5% CO_2_, pH 7.4) for 30 min prior whole-cell recordings.

Differential interference contrast optics was used to visualize neurons using an upright Nikon PERlipse microscope (Nikon, Germany) coupled to a 530 nm high power LED collimator source (Mightex Systems, Canada). Whole-cell patch clamp recordings were made at 30 °C in aCSF containing normal MgCl_2_ (1 mM) and CaCl_2_ (2 mM). Patch electrodes were made from borosilicate glass (2–3 M∧) filled with a current-clamp high K^+^ intracellular solution (composition in mM: 110 K^+^-Gluconate; 30 KCl; 10 Hepes; 10 Na_2_phosphocreatine; 0.2 EGTA; 2 Mg-ATP; 0.5 Li-GTP; 1 MgCl_2_; pH was adjusted to 7.3 with KOH). No spontaneous action potentials discharge was observed at the resting membrane potential. Despolarizing direct current injection (50–150 pA) was delivered in order to generate tonic action potentials. Electrical signals were recorded using an AxoClamp 200B amplifier commanded by pCLAMP 10.0 software (Molecular Devices, CA, USA). Data were filtered at 5 kHz, digitized and stored for off-line analysis using Clampfit. CNO was prepared as a stock solution in DMSO and aliquots were stored at −20 °C. CNO aliquots were unfrozen fresh every day and locally delivered using a Picospritzer II (General Valve Corporation, Fairfield, NJ) coupled to a *puff* glass pipette filled with CNO (100 µM in normal aCSF, 1% DMSO) and located at distance of ~50 μm from the recorded mCherry expressing-patch-clamped cortical pyramidal neuron.

### Preclinical Positron Emission Tomography imaging

#### Animal handling

Four h starved mice were IP injected with 25 µCi/g [18F]-FDG at different times prior to- or after the beginning of the re-exposure to the training context. Thirty min before [18F]-FDG injection and during the first stage of the radiopharmaceutical incorporation, while waiting for PET acquisition, mice were left undisturbed in individual home cages in a 29 °C environment, to which they habituated 2 days before the experiments. In order to optimize the regional [18F]-FDG contrasts in relation to basal conditions, all protocols were designed so that the behavioral moment of interest for glucose consumption analyses occurred in concordance with the maximum radiotracer availability on plasma, which was reported to be 10 min after [18F]-FDG IP injection^34^. For PET acquisition, mice were anesthetized under a mix of isoflurane (4.5% for induction and 1.5% for maintenance) and O_2_ for approximately 2–3 min and then put in the tomograph, which maintained their body temperature at 35 °C during the acquisition. After acquisition, mice were returned to their home cages, and in some experiments were tested for long-term contextual memory 48 h after TR.

#### Imaging system

Images were acquired using a preclinical PET TriFoil LabPET 4 with LYSO and GYSO crystals and 1536 APD detectors groups. Approximated spatial resolution FWHM = 1.2 mm (full width at half maximum), 3.7 cm axial and 11 cm trans-axial field of view (FOV).

#### Acquisition and reconstruction setup

Each PET acquisition lasted 12 min -list-mode acquisition-. Images were reconstructed using OSEM 3D algorithm with 30 iterations, by which the best signal to noise ratio was achieved. If motion was detected during acquisition, dynamic reconstruction was performed in order to correct it using SPM5 on MATLAB® realign algorithm.

#### Image spatial processing

The images were confined to a bounding-box only including the brain. A normal subject-based template was created in order to have an anatomic reference for realignment and normalization. All images were smoothed using an isotropic Gaussian kernel with 1 mm FWHM. Images were co-registered and normalized to this template using SPM5 on MATLAB®, with the following parameters: normalized mutual information as objective function and 7 mm smoothing histogram for rigid co-registration; affine regularization to the averaged template size, no-smooth and 2–0.1 mm of separation for the non-rigid normalization. Previous to intensity normalization and statistical analysis, a brain masking avoiding Harderian glands was applied to all subjects, since the uptake in these glands can significantly modify the intensity normalization values (data not shown).

### *In vivo* electrophysiology and optogenetics

Adeno-associated viral vector carrying ChR2 fused to GFP, under the control of the CAG promoter was unilaterally injected into the left lateral neocortex (same coordinates as in chemogenetics section). Recordings were made using a drivable electrode array (Innovative Neurophysiology Inc.) implanted unilaterally together with an optic fiber in the left amygdala. Coordinates were 1.5 mm anteroposterior from bregma; 3.3 mm left from midline; 3.7 mm ventral from brain surface. After surgery, electrodes downed further 0.5 mm, to finally reach the intended site. For optical stimulation, we applied blue light pulses of 10 msec, 1 Hz. In the case of paired pulse experiments, 10 msec blue light pulses were separated with intervals of 100, 40 and 20 msec. Data was processed with commercial software (Offline Sorter and Neuroexplorer; Plexon). After experiments mice were deeply anesthetized and perfused first with saline and then with PFA 4%. Brains were post-fixed in PFA overnight and remained in PBS 30% sucrose until used. Coronal sections were checked for fluorescence in the lateral neocortex and the correct placement of electrodes.

### Morphologic studies

We performed neuroanatomical retrograde tracing according to Conte *et al*.^85^. Briefly, mice were anesthetized as mentioned before and were injected with 0.12 µl of CTB conjugated with Alexa Fluor-594 (Invitrogen, cat. no. C22842) 0.5% w/v diluted in saline, into the left amygdala. Coordinates were: 1.6 mm anteroposterior from bregma; 3.3 mm left from midline; 4.9 mm ventral from bregma. Seven days after injection we perfused the mice first with saline and then with PFA 4%. Brains were post-fixed in PFA overnight and remained in PBS 30% sucrose until used. Coronal sections were prepared for fluorescence analysis. Fluorescence images were taken with confocal microscope/episcope depending on the magnification, and brightness/contrast was modified to better visualize marked cells.

### Experimental design and statistical analysis

#### Behavioral experiments

Numbers of mice (n) in each group are shown in Figure Legends. All data are shown as mean ± SEM. Freezing scores were analyzed by General Linear Models (GLM) and Akaike information criterion was considered in order to choose the best variance structure. LSD comparison test was performed to compare data between groups. To test for homocedasticity and normality assumptions, we considered residual dispersion graphs together with Levene and Shapiro-Wilks tests, respectively (InfoStat 2016. Grupo InfoStat, FCA, Universidad Nacional de Córdoba, Argentina; R Foundation for Statistical Computing, Vienna, Austria). When more than one behavioral test was performed to all the groups in one experiment, data were analyzed as repeated measures. When the experiment included more than one treatment and more than one behavioral test, which was performed to some of the groups in one experiment but not to all (i.e. Non re-exposed animals are not evaluated in re-exposure day), we analyzed data of each day separately. We performed post-hoc contrasts corrected for multiple comparisons (Sidak). A significance level of P < 0.05 was used for all behavioral analyses.

#### PET Image statistical analyses

All groups of subjects were analyzed as a multi-factorial ANOVA test using SPM5 on MATLAB®. Intensity normalization was considered as a regressor variable for each factor using grand mean scaling and ANCOVA. Global calculation of individual means was calculated over each masked brain. In order to have an accurate anatomical reference, all results of statistical differences where co-registered with an MRI atlas^86^. Spatial transformation was applied to the MRI atlas to correct for the differences between mice strains and methodological animal. Only brain areas showing statistical differences in glucose consumption between groups in two or more replicates of the experiments were considered throughout the paper.

### Significant statement

The ability to learn from environmental stimuli is universal the animal kingdom and it enables to develop adaptive behaviors. Consequently, the ability to remember what has been learnt has a relevant role in every animal’s life. Besides, cognitive function is a product of the concerted and synchronized activity of many brain regions. However, the study of the physiology underlying memory in small animals using whole-brain techniques is scarce. Using Positron Emission Tomography and chemogenetics, we show that the lateral neocortex is necessary for fear reconsolidation, a mnemonic process that enables strengthening and updating of memory. Inhibition of lateral neocortex during reconsolidation changed glucose consumption levels in the amygdala, a key region for emotional memories, where the lateral neocortex projects to.

## Supplementary information

Supplementary Information
