# Supplementary material for: The lateral neocortex is critical for contextual fear memory reconsolidation
Source: Sci Rep. 2019 Aug 21;9:12157. doi: 10.1038/s41598-019-48340-9 (PMC6704072; doi:10.1038/s41598-019-48340-9)

Supplementary Information

Title: The lateral neocortex is critical for contextual fear memory reconsolidation.

Authors: Verónica de la Fuente[1,2*#], Candela Medina[1,2#], Germán Falasco[3], Leandro Urrutia[3], Alexxai V. Kravitz[4], Francisco J. Urbano[1,2], Silvia Vázquez[3], María Eugenia Pedreira[1,2] and Arturo Romano[1,2].

Affiliations:

[1]Universidad de Buenos Aires, Facultad de Ciencias Exactas y Naturales, Departamento de Fisiología, Biología Molecular y Celular, Buenos Aires, Argentina.

[2]CONICET-Universidad de Buenos Aires, Instituto de Fisiología, Biología Molecular y Neurociencias (IFIBYNE), Buenos Aires, Argentina.

[3]Centro de Imágenes Moleculares, Fundación para la Lucha contra las Enfermedades Neurológicas de la Infancia (FLENI), Escobar, Buenos Aires, Argentina.

[4]National Institute of Diabetes and Kidney and Digestive Diseases, Bethesda, MD 20814, USA.

***Corresponding author:** verodelaf@gmail.com

[#] Both authors contributed equally to this work

Figure Legends

Supplementary Figure 1

A) Experimental protocol designed to measure glucose consumption. We injected mice [18F]-FDG IP 10 min pre re-exposure (R5: re-exposed 5 min; R1: re-exposed 1 min), and after re-exposure we anesthetized them with isoflurane and analyzed with PET (for simplicity both anaesthesia and acquisition of images with PET are altogether indicated as "PET scan"). Non-re-exposed mice (NR) were injected with [18F]-FDG 24 h post training. n = 11 - 12. TR: training; Re-exp: context re-exposure. Experimental procedures in day 2 are shown in grey. **B, C, D, E)** Small-animal PET images corresponding to different coronal sections across the entire mouse brain. Data were analyzed using SPM ANOVA design and normalized through ANCOVA regressors. Pair-wise comparisons yielding P values <0.01 are shown using a t statistic color scale, which corresponds to the level of significance at the voxel level. Images are displayed with the left side corresponding to the left hemisphere, according to neurological conventions. **B)** R5 vs NR groups; **C)** R5 vs R1 groups; **D)** R1 vs NR groups; **E)** Naive vs NR groups.

Supplementary Figure 2

A) Similar to Supplementary Fig. 1 but injecting [18F]-FDG at different time points to study dynamics of glucose consumption in animals that express and labilizate/reconsolidate memory. [18F]-FDG was injected either immediately before 5 min re-exposure or 35 min after its offset (R5-pre and R5-35min, respectively). The third group was not re-exposed (NR)

but received FDG injection 24 h after training and served for comparisons. Time interval between [18F]-FDG administration and anesthesia was 20 min (for simplicity, both anaesthesia and acquisition of images with PET are altogether indicated as “PET scan”). n = 12. TR: training; Re-exp: context re-exposure. Experimental procedures in day 2 are shown in grey. **B, C)** Small-animal PET images corresponding to different coronal sections across the entire mouse brain. Data were analyzed using SPM ANOVA design and normalized through ANCOVA regressors. Pair-wise comparisons yielding P values <0.01 are shown using a t statistic color scale, which corresponds to the level of significance at the voxel level. Images are displayed with the left side corresponding to the left hemisphere, according to neurological conventions. **B)** R5pre vs NR groups; **C)** R5-35 vs NR groups.

Supplementary Figure 3

A) We injected [18F]-FDG 35 min after the offset of a 1 min re-exposure to the TR context (R1-35min). Control group was not re-exposed (NR) but received FDG injection 24 h after training and served for comparisons. Time interval between [18F]-FDG administration and anesthesia was 20 min (for simplicity, both anaesthesia and acquisition of images with PET are altogether indicated as “PET scan”). n = 11-12. TR: training; Re-exp: context re-exposure. Experimental procedures in day 2 are shown in grey. **B)** Small-animal PET images corresponding to different coronal sections across the entire mouse brain. Data were analyzed using SPM ANOVA design and normalized through ANCOVA regressors. Pair-wise comparisons yielding P values <0.01 are shown using a t statistic color scale, which

corresponds to the level of significance at the voxel level. Images are displayed with the left side corresponding to the left hemisphere, according to neurological conventions.

Supplementary Figure 1

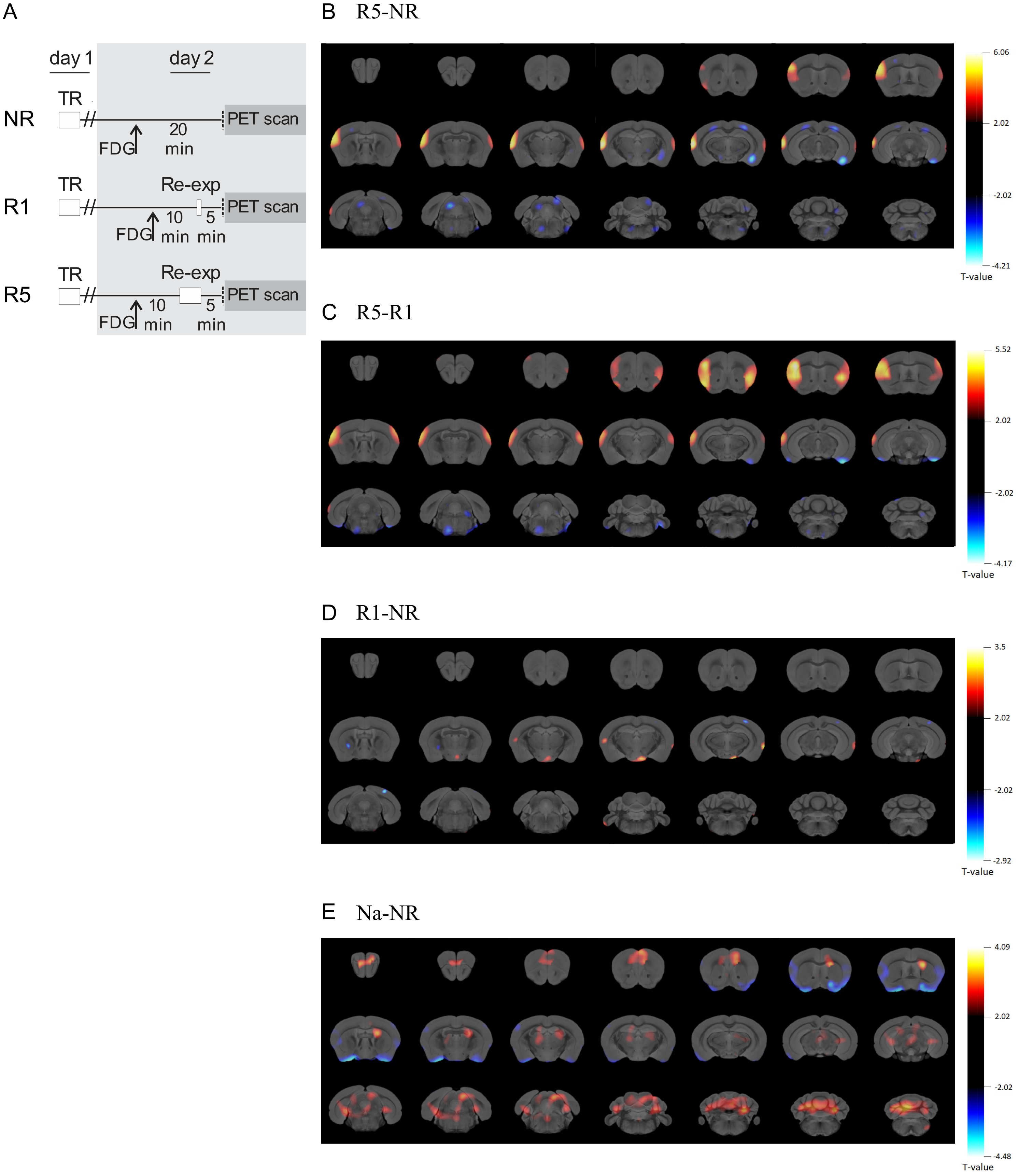

Supplementary Figure 2

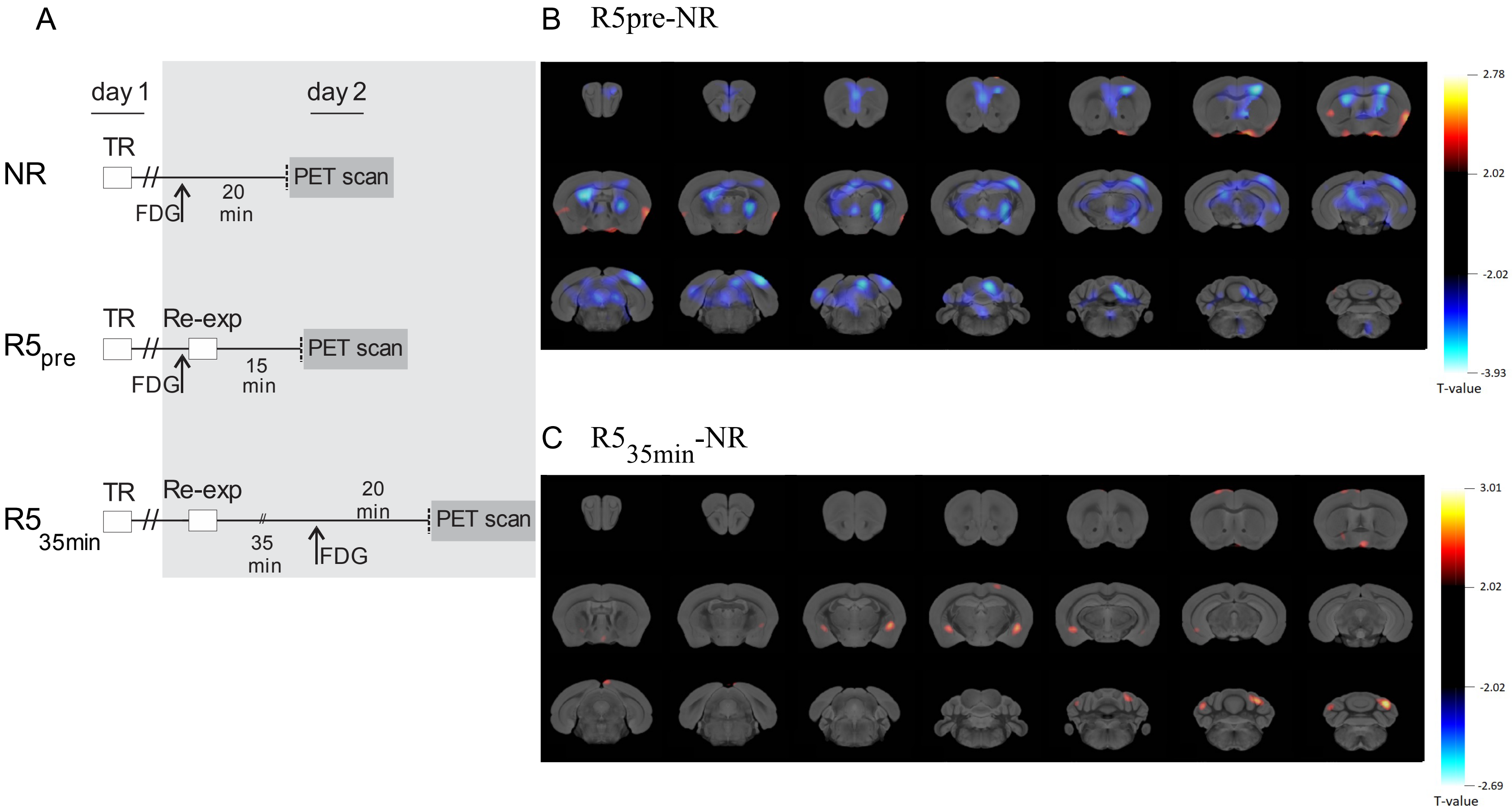

Supplementary Figure 3

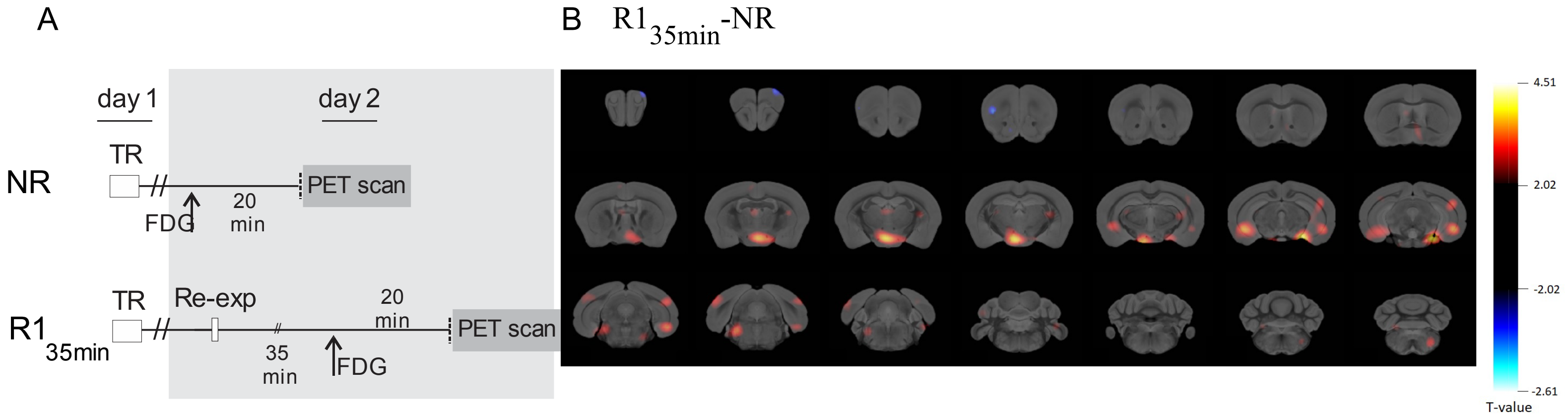

Supplement: Supplementary file 1 — Supplementary Information [file 41598_2019_48340_MOESM1_ESM.pdf]
